# Comparison of the aggregation of homologous β_2_-microglobulin variants reveals protein solubility as a key determinant of amyloid formation

**DOI:** 10.1016/j.jmb.2016.01.009

**Published:** 2016-02-13

**Authors:** Clare L. Pashley, Eric W. Hewitt, Sheena E. Radford

**Affiliations:** Astbury Centre for Structural Molecular Biology, School of Molecular and Cellular Biology, University of Leeds, Leeds, LS2 9JT, UK

**Keywords:** AUC, analytical ultracentrifugation, ANS, anilino-1-naphthalenesulfonate, β_2_m, β_2_-microglobulin, C_s_, critical concentration, CD, circular dichroism, DRA, dialysis related amyloidosis, EM, electron microscopy, *h*β_2_m, human β_2_-microglobulin, HSQC, heteronuclear single quantum coherence spectroscopy, *m*β_2_m, mouse β_2_-microglobulin, NMR, nuclear magnetic resonance, ThT, thioflavin T, Aggregation, Amyloidogenicity, Critical concentration, Solubility, Amyloid kinetics

## Abstract

The mouse and human β_2_-microglobulin protein orthologs are 70 % identical in sequence and share 88 % sequence similarity. These proteins are predicted by various algorithms to have similar aggregation and amyloid propensities. However, whilst human β_2_m (*h*β_2_m) forms amyloid-like fibrils in denaturing conditions (e.g. pH 2.5) in the absence of NaCl, mouse β_2_m (*m*β_2_m) requires the addition of 0.3 M NaCl to cause fibrillation. Here, the factors which give rise to this difference in amyloid propensity are investigated. We utilise structural and mutational analyses, fibril growth kinetics and solubility measurements under a range of pH and salt conditions, to determine why these two proteins have different amyloid propensities. The results show that, although other factors influence the fibril growth kinetics, a striking difference in the solubility of the proteins is a key determinant of the different amyloidogenicity of *h*β_2_m and *m*β_2_m. The relationship between protein solubility and lag time of amyloid formation is not captured by current aggregation or amyloid prediction algorithms, indicating a need to better understand the role of solubility on the lag time of amyloid formation. The results demonstrate the key contribution of protein solubility in determining amyloid propensity and lag time of amyloid formation, highlighting how small differences in protein sequence can have dramatic effects on amyloid formation.

## Highlights

• Human and murine β_2_m share high sequence homology.
• Human β_2_m forms amyloid fibrils more readily than murine β_2_m at low pH.
• Protein solubility is a key determinant of the initiation of amyloid formation.
• Greater understanding of protein solubility may enhance predictions of lagtime.

## Graphical abstract

**Figure:**
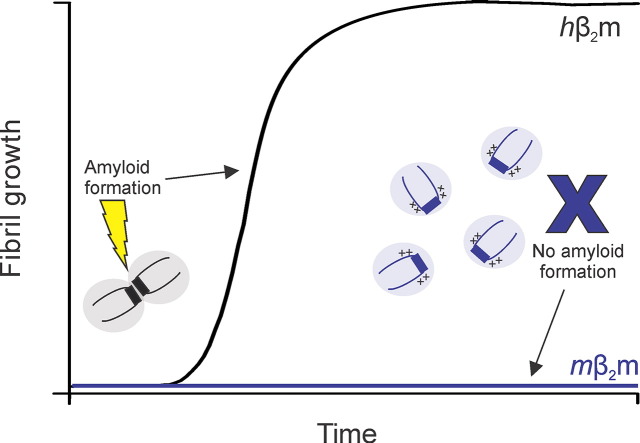

## Introduction

**Fig. 1:**
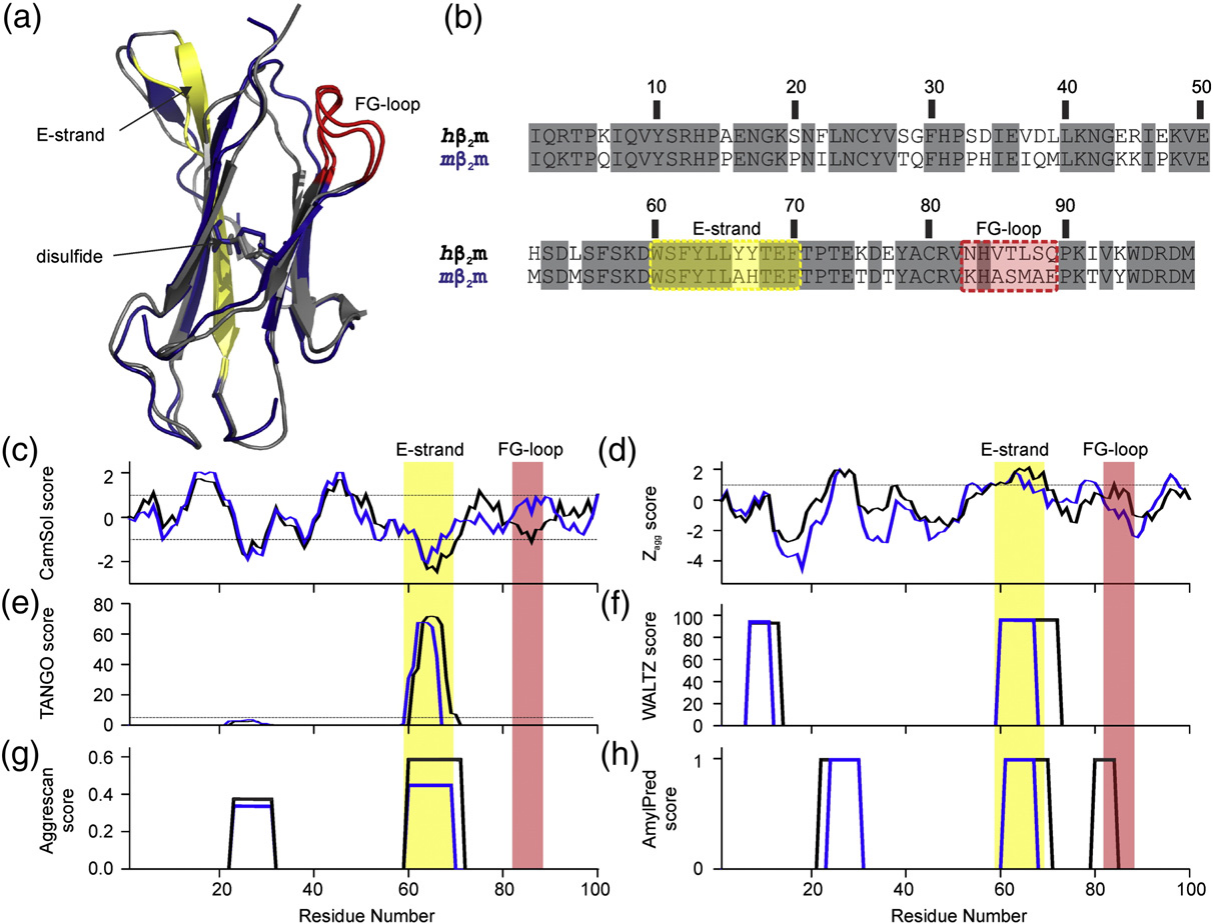
**Human and mouse β_2_m have high sequence and structural similarity**. (a) Ribbon diagram showing the structural alignment of *h*β_2_m (PDB: 2XKS [10]), and *m*β_2_m (PDB: 1LK2 [51]) crystal structures, coloured in *grey* and *blue*, respectively. The E-strand is coloured in *yellow* and the FG-loop in *red* in each structure. The figure was created using PyMol [52]. (b) Sequence alignment of *h*β_2_m and *m*β_2_m with the E-strand and the FG loop highlighted in pale *yellow* and *red*, respectively. Identical residues are boxed in grey. (c) CamSol solubility prediction profiles at pH 2, with threshold values for soluble (+ 1) and insoluble (-1) regions shown [11]; (d) Zyggregator amyloid propensity prediction profiles for pH 2, with the threshold value for aggregation-prone regions shown (≥ 1) [14]; (e) TANGO aggregation prediction profiles for pH 2, indicating the percentage likelihood of a region being aggregation-prone, with the threshold value (> 5 %) for aggregation-prone sequences shown [53]; (f) WALTZ amyloid prediction profiles for pH 2.6, indicating the percentage likelihood of a sequence being able to form amyloid [13]; (g) Aggrescan aggregation profiles indicating the “normalised hotspot area” for aggregation-prone regions [12]; and (h) AmylPred consensus profile where regions with a value of + 1 are predicted to have a high propensity to form amyloid. The algorithm parameters closest matching the low pH fibril growth buffer were chosen where possible. In c-h *h*β_2_m is in black and *m*β_2_m is in blue.

β_2_-microglobulin (β_2_m) is the light chain of the class I major histocompatibility complex [1]. This 99-residue protein (Fig. 1a) is associated in humans with dialysis-related amyloidosis (DRA) and in rare instances, familial amyloidosis [1], [2], [3], [4]. In healthy individuals human β_2_m (*h*β_2_m) is degraded and excreted by the kidneys; however β_2_m concentrations increase more than 50-fold in the plasma of patients on long-term hemodialysis [1]. This increase in β_2_m concentration is thought to facilitate aggregation of *h*β_2_m into amyloid fibrils, which form systemic deposits, most notably in the osteoarticular tissues [1], [2], [3], [4], [5], [6], [7], [8], [9], [10]. In mice, the serum levels of β_2_m are 100-times higher than in healthy humans, and > 5 times higher than in humans on dialysis [4]. However, despite these high serum concentrations, amyloid deposits of β_2_m are not observed in mice [4]. This striking difference in the behaviour of β_2_m *in vivo* appears counterintuitive as the mouse β_2_m (*m*β_2_m) and *h*β_2_m sequences are 70 % identical, share 88 % sequence similarity and the native proteins have very similar structures (Fig. 1a,b).

Several algorithms predict the propensity for proteins to aggregate and/or form amyloid based on the protein sequence alone [11], [12], [13], [14], [15], [16]. These prediction algorithms (Fig. 1c-h) highlight the E-strand of β_2_m as either being one of the most, or the most, aggregation-prone region(s) of the protein. Only minor differences between the predictions for the *m*β_2_m and *h*β_2_m sequences are identified, consistent with the high sequence similarity of the proteins (Fig. 1 c-h). Yet despite the predicted amyloidogenicity of *h*β_2_m, monomeric *h*β_2_m does not form amyloid fibrils *de novo* at neutral pH *in vitro*, without the addition of cofactors such as heparin, SDS, or Cu^2 +^ or by the truncation of the protein [2], [7], [9], [10]. However, upon unfolding by acid denaturation and agitation, *h*β_2_m rapidly forms fibrils with a parallel in register cross-beta structure typical of amyloid [17], [18]. Since the aggregation propensity for the human and mouse β_2_m sequences is predicted to be similar it would be expected that *m*β_2_m, like *h*β_2_m, would also aggregate when acid unfolded. Surprisingly, *in vitro* studies have shown that *m*β_2_m does not form fibrils under these conditions (i.e. low pH with agitation) [4], [10], [19].

Since the extent of the difference in amyloidogenicity of *m*β_2_m and *h*β_2_m is so marked and different prediction methods fail to capture this difference (Fig. 1c-h), these sequences provide an ideal model to interrogate the key determinants of amyloidogenicity [4], [10], [19]. Here, the conformational properties and sequence changes that give rise to the observed difference in amyloid propensity between human and mouse β_2_m are analysed through structural and mutational analyses, fibril growth kinetics and solubility measurements. The results demonstrate that small sequence changes have a dramatic effect on the solubility and the lag time of amyloid formation in these acid-unfolded proteins. The results add to a growing body of evidence indicating that protein solubility is an important determinant of amyloid propensity even in the absence of structural factors [11], [14], [20], [21], [22], [23], [24], [25], [26], [27], [28], [29], [30], [31], [32], [33], [34], [35]. Importantly, current algorithms are unable to reliably predict the differences in lag time of fibril formation observed for the *m*β_2_m and *h*β_2_m sequences. Improving our ability to predict the effect of residues flanking amyloid-prone regions on aggregation, and how sequence differences affect solubility, may allow for a greater understanding of the lag time of amyloid formation and how protein variants associated with disease cause protein aggregation *in vivo*.

## Results

### Ionic strength and the aggregation of *m*β_2_m

**Fig. 2:**
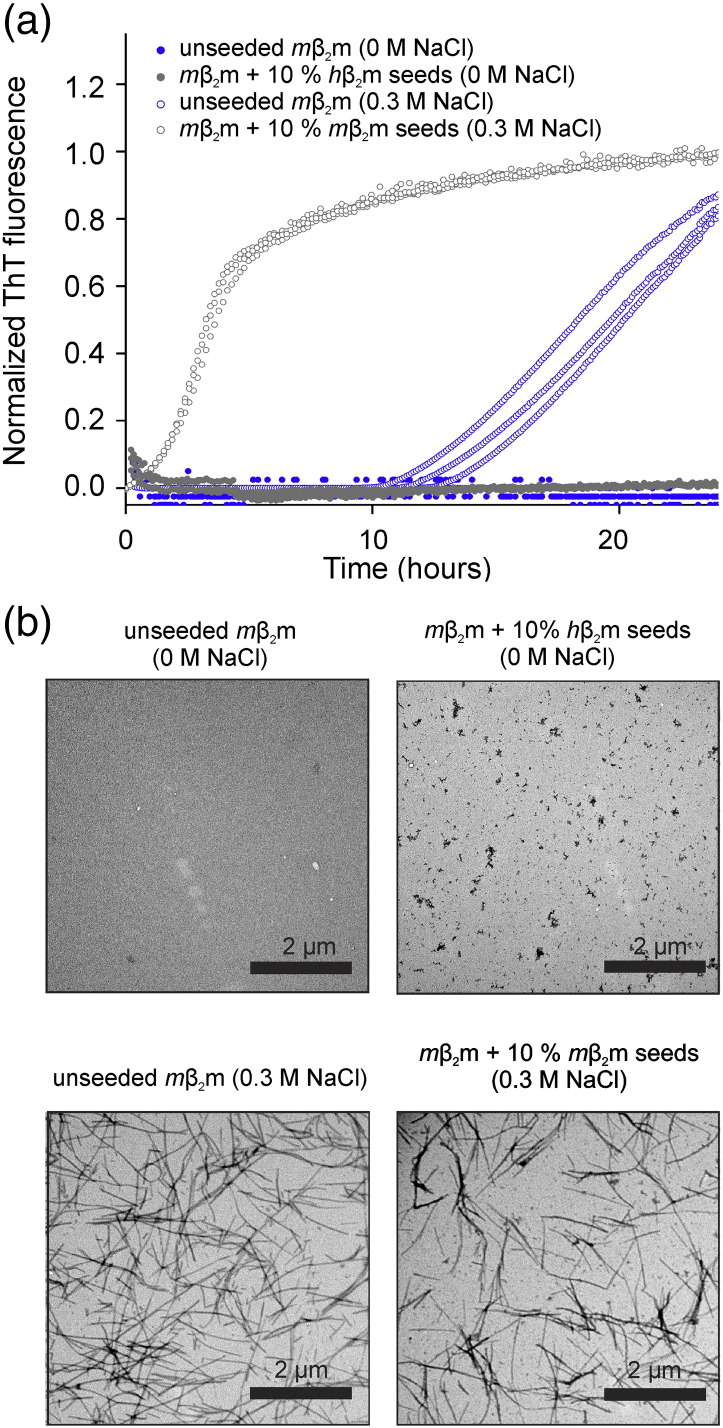
***m*β_2_m forms fibrils in the presence of NaCl**. Fibril growth kinetics at pH 2.5 of 50 μM *m*β_2_m agitated at 200 rpm and monitored by ThT fluorescence in the absence (*closed* data points) or presence of 0.3 M NaCl (*open* data points). Unseeded reactions are coloured *blue*. Seeded growth experiments (*grey*) were performed in 0 M NaCl with 10 % (v/v) *h*β_2_m fibril seeds formed in fibril growth buffer (pH 2.5) with 0 M NaCl, or in 0.3 M NaCl with 10 % (v/v) *m*β_2_m fibril seeds formed in fibril growth buffer with 0.3 M NaCl. Three replicates of each condition are shown. (b) The endpoint negative stain EM images of the above reactions after 24 hours incubation are shown as indicated (see Materials and Methods).

Previous studies of acid-unfolded *m*β_2_m showed that the protein is unable to form amyloid fibrils by quiescent incubation, even in the presence of 1.5 M NaCl (in 25 mM sodium phosphate, pH 2.0 at 37 °C) [4]. By contrast, *h*β_2_m forms fibrils within 3 hours under identical conditions [4]. Here, to investigate the amyloidogenicity of *m*β_2_m and *h*β_2_m in more detail, *m*β_2_m was incubated in pH 2.5 fibril growth buffer (25 mM sodium phosphate/25 mM sodium acetate) at 37 °C and agitated at 200 rpm (Fig. 2a) [36], [37], [38]. Consistent with previous results [4], [10], [19], *m*β_2_m did not form fibrils under these conditions (Fig. 2a). Furthermore, addition of 10 % (*v*/*v*) *h*β_2_m fibril seeds formed at pH 2.5 (see Materials and Methods [36], [39]) did not induce fibril growth (Fig. 2a). By contrast with these results, in the presence of 0.3 M NaCl and agitation at 200 rpm fibril growth was observed for *m*β_2_m at pH 2.5 (Fig. 2a) with the lag time being reduced upon the addition of 10 % (w/w) fragmented *m*β_2_m fibrils formed in the presence of 0.3 M NaCl, consistent with a seeding reaction (Fig. 2a). Interestingly *m*β_2_m fibrils did not seed in 0 M NaCl, pH 2.5 and *h*β_2_m seeds did not initiate *m*β_2_m fibrillation in 0.3 M NaCl, pH 2.5 (*data not shown*). Negative-stain EM images confirmed the presence of amyloid-like fibrils of *m*β_2_m in the presence of 0.3 M NaCl, but not in the absence of NaCl (Fig. 2b), confirming the requirement of higher ionic strength conditions to facilitate self-assembly of *m*β_2_m even when the protein is unfolded at acidic pH.

**Fig. 3:**
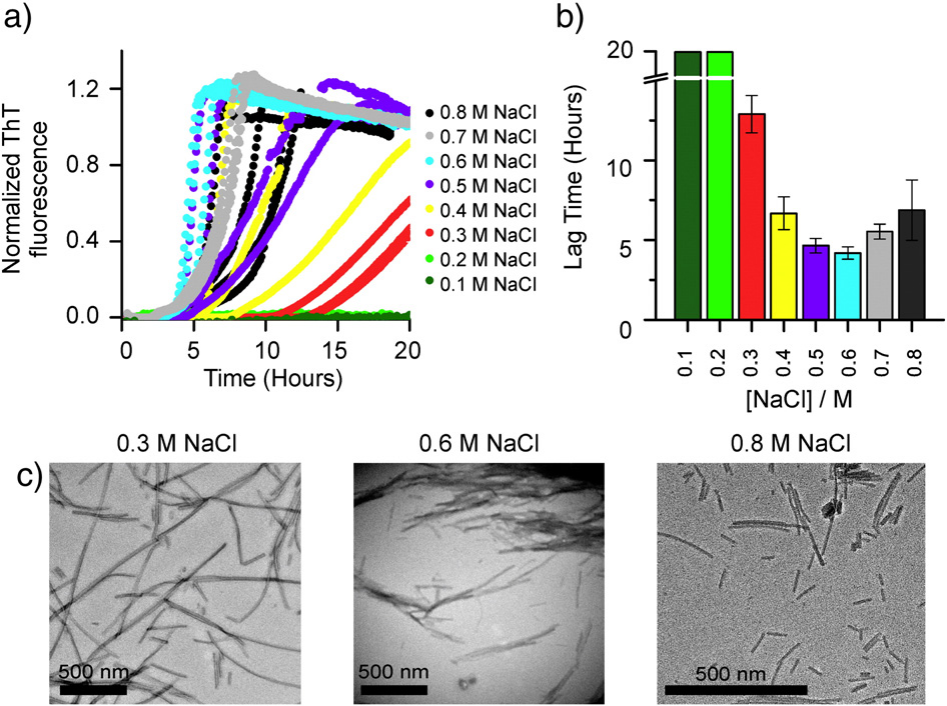
**Fibrillation of *m*β_2_m is dependent on NaCl concentration**. (a) Fibril growth kinetics of 50 μM *m*β_2_m agitated at 200 rpm and monitored by ThT fluorescence at pH 2.5 in the presence of 0.1 - 0.8 M NaCl. (b) Lag time analysis of the fibril growth curves shown in (a). In cases where no fibrils were observed the lag time is plotted with a value of 20 hours as a lower limit for an indefinite lag time. (c) Negative-stain EM images after 24 hours incubation of *m*β_2_m in pH 2.5 fibril growth buffer with 0.3 M NaCl, 0.6 M NaCl or 0.8 M NaCl, as indicated. The scale bar for 0.8 M NaCl is different to that of 0.3 and 0.6 M NaCl because the fibrils were considerably smaller.

Next, a systematic investigation of the effect of ionic strength on fibril formation by *m*β_2_m was performed (Fig. 3a). As observed previously for acid unfolded *h*β_2_m [38], [40], there is an optimum NaCl concentration at which the shortest lag time for fibril growth occurs (0.6 M NaCl for *m*β_2_m at pH 2.5) (Fig. 3b) [40]. As Yoshimura *et al.* noted for *h*β_2_m, this is most likely due to a charge-balance requirement for fibril formation: electrostatic repulsion must be overcome in order for the protein subunits to interact, but too many interactions can trap the protein in off-pathway soluble oligomers or amorphous insoluble aggregates slowing down amyloid formation. This rationale is supported by the observation that *m*β_2_m forms abundant short fibrils at ionic strengths > 0.6 M (Fig. 3c). The fibrils of *m*β_2_m formed in 0.3 M NaCl were the longest and most dispersed of all conditions investigated (Fig. 3c). Thus, this salt concentration was chosen for further experiments.

### Conformational properties of denatured β_2_m and the determinants of amyloidogenicity

A contributing factor to the different amyloid propensities of *m*β_2_m and *h*β_2_m could be differences in their conformational properties at pH 2.5. To compare the conformations of the acid denatured states of these proteins CD, ^1^H- ^15^N HSQC NMR spectra and the binding of anilino-1-naphthalenesulfonate (ANS) to each protein were assessed at pH 2.5 (SI Fig. 1a-f). The far-UV CD spectra show that both proteins are highly unfolded at pH 2.5 (SI Fig. 1a and b), consistent with the limited ^1^H dispersion observed in the ^1^H- ^15^N HSQC spectra of each protein which is also indicative of an unfolded protein ensemble (SI Fig. 1c and d). Interestingly, the far-UV CD spectra of *m*β_2_m and *h*β_2_m are different. This is likely due to differences in the aromatic contributions to the spectra of the native proteins at neutral pH and subtle differences in the populations of species in the unfolded ensembles at pH 2.5 (SI Figs. 1a and b). ANS fluorescence emission spectra show no increase in fluorescence intensity and no change in the fluorescence emission λ_max_ for *m*β_2_m and *h*β_2_m in 0.3 M NaCl compared with 0 M NaCl, ruling out substantial differences in the distribution of solvent exposed hydrophobic patches in the two acid unfolded proteins (SI Fig. 1e). Furthermore, even in the presence of NaCl no significant conformational changes were detected for the *m*β_2_m acid unfolded ensemble, as detected by ANS binding and far-UV CD (SI Fig. 1e and f). Together these results show that gross differences in the residual structure of acid denatured *m*β_2_m and *h*β_2_m are unlikely to be responsible for the differences in their amyloid propensity observed.

There are fewer peaks in the ^1^H- ^15^N HSQC spectrum of *h*β_2_m in pH 2.5 buffer compared with that of *m*β_2_m under the same conditions (SI Fig. 1c and d). The line broadening that causes the loss of peaks in the ^1^H- ^15^N HSQC spectrum of *h*β_2_m may indicate conformational exchange on the micro- to millisecond timescale and/or oligomerization of the protein, which could be linked to the increased aggregation propensity of *h*β_2_m. To investigate the relationship between line broadening and amyloidogenicity, a series of ^1^H- ^15^N HSQC spectra were acquired. Spectra of *m*β_2_m were recorded in pH 2.5 water, 25 mM sodium phosphate/ 25 mM sodium acetate (pH 2.5), and the same buffer containing either 0.3 M or 0.8 M NaCl (SI Fig. 2). The spectra show that as the salt concentration is increased the line broadening is also increased and peaks are lost from the spectra. In 0.8 M NaCl only 20 of the total 73 peaks are visible (SI Fig. 2d). Since these peaks reappear with the addition of 8 M urea this is not a consequence of the high salt conditions affecting data acquisition (SI Fig. 3). To investigate whether the oligomerization of the protein contributes to the line broadening, sedimentation velocity analytical ultracentrifugation (AUC) was performed on the samples (SI Fig. 4). These results revealed that *m*β_2_m is predominantly a single species at pH 2.5, with a weight-averaged *S*_20,w_ value of 1.23S, consistent with a monomeric species of β_2_m [41]. However, higher order species are observed in 0.3 M NaCl and 0.8 M NaCl. In 0.8 M NaCl the protein is predominantly dimeric and in higher order oligomers (SI Fig. 4), consistent with the line broadening observed in the NMR spectra obtained in 0.8 M NaCl (SI Fig. 2d). These results suggest that increased concentrations of NaCl favour aggregation, at least in part, by increasing the likelihood of the formation of oligomers that can initiate aggregation.

### Sequence determinants of amyloidogenicity

The E-strand is the most amyloidogenic β-strand of the β_2_m sequence [36], [42]. There are two differences between the sequence of *m*β_2_m and that of *h*β_2_m in this region (Y66 and Y67 in *h*β_2_m, A66 and H67 in *m*β_2_m) (Fig. 1b). These substitutions result in differing aggregation propensity for this region, with *m*β_2_m predicted to be marginally less aggregation prone than its human counterpart using three of the aggregation prediction algorithms tested (Fig. 1d,e,g). To determine whether the difference in amyloid propensity of *m*β_2_m and *h*β_2_m is related to the sequence differences in this region, the E-stand of both β_2_m variants were synthesised as peptides. Incubation of these peptides at pH 2.5 resulted in fibril formation of both peptides immediately upon dilution out of DMSO into fibril growth buffer (*data not shown*). Fibrils were confirmed by negative-stain transmission EM (SI Fig. 5), ruling out sequence differences in this region as the major contributing factor to the very different amyloid propensities of their parent protein sequences.

**Fig. 4:**
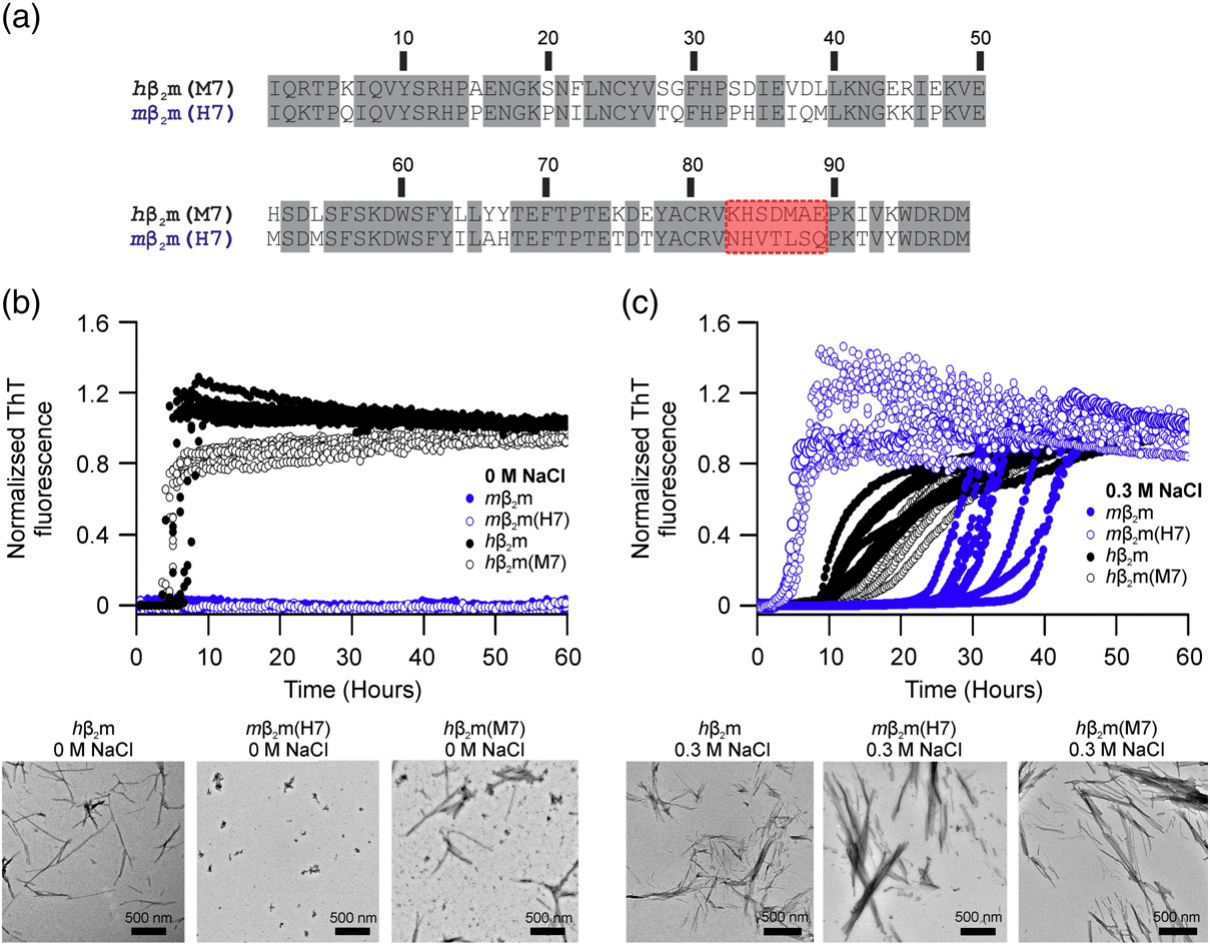
**Sequence determinants of β_2_m amyloidogenicity**. (a) Sequence alignment of the chimeric proteins *h*β_2_m(M7) and *m*β_2_m(H7) in which regions 83-89 of *h*β_2_m have been replaced with the corresponding residues in *m*β_2_m and *vice versa*. The regions that are switched are shown in red (b) Fibril growth kinetics of 50 μM protein agitated at 200 rpm monitored by ThT fluorescence at pH 2.5 in (b) 0 M or (c) 0.3 M NaCl. Data for *m*β_2_m, *m*β_2_m(H7), *h*β_2_m and *h*β_2_m(M7) are shown as *closed* blue, *open* blue, *closed* black and *open* black data points, respectively. End-point negative stain EM images of *h*β_2_m, *m*β_2_m(H7) and *h*β_2_m(M7) in 0 M or 0.3 M NaCl are shown in the lower panels of (b) and (c), respectively. EM images of *m*β_2_m in 0 M NaCl or 0.3 M NaCl are shown in Fig. 2b.

Previously Ivanova *et al.* attributed the differences in amyloidogenicity of *m*β_2_m and *h*β_2_m to the FG loop region [4]. The group made chimeric proteins, in which the FG loop sequence (residues 83-89) of *h*β_2_m was replaced by the corresponding region from *m*β_2_m to create *h*β_2_m(M7). The complementary protein *m*β_2_m(H7) was generated by replacing residues 83-89 of *m*β_2_m with the corresponding *h*β_2_m sequence (Fig. 4a). Their analysis of the chimeric proteins revealed that *h*β_2_m(M7) aggregated more slowly than *h*β_2_m at pH 2.0 under quiescent conditions in 200 mM NaCl. Conversely, *m*β_2_m(H7) formed fibrils, whereas the wild-type *m*β_2_m sequence did not [4]. We analysed the aggregation kinetics of *h*β_2_m(M7) and *m*β_2_m(H7) at different ionic strengths at pH 2.5 (Fig. 4b and c). No fibril growth for *m*β_2_m or *m*β_2_m(H7) was observed in 0 M NaCl after incubation and shaking (200 rpm) for 60 hours (Fig. 4b). By contrast, the *h*β_2_m(M7) aggregated at a similar rate as its *h*β_2_m counterpart in 0 M NaCl (lag times 4.2 ± 1.1 hours, 5.5 ± 1.1 hours, respectively) (Fig. 4b, Table 1). In 0.3 M NaCl all four proteins formed fibrils within 60 hours, although *h*β_2_m(M7) aggregated more slowly than *h*β_2_m (lag time of 13.7 ± 1.5 hours, 9.2 ± 0.6 hours, respectively) (Fig. 4c). In 0.3 M NaCl, a dramatic effect on the behaviour of *m*β_2_m(H7) was observed, in which the lag time is reduced substantially when compared with *m*β_2_m (lag times 4.1 ± 0.5 hours, 30.1 ± 4.5 hours, respectively) (Fig. 4c, Table 1). This striking 7-fold reduction in lag time confirms that the FG loop region of *h*β_2_m is responsible, at least in part, for the enhanced amyloid propensity of the *m*β_2_m(H7) sequence. Interestingly, the lack of such a dramatic effect in the *h*β_2_m(M7) sequence compared with *h*β_2_m indicates that the effect is dependent both on the sequence of the FG loop and that of the host protein.

**Fig. 5:**
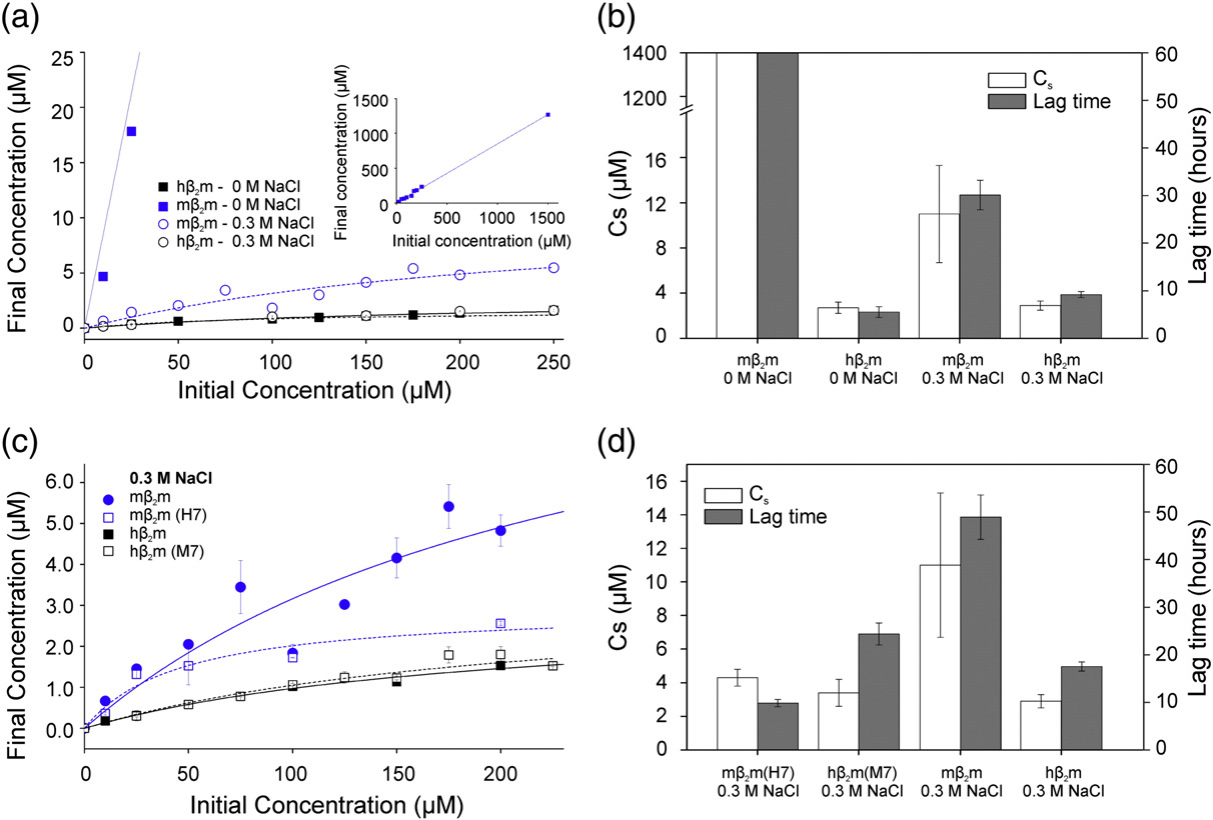
***m*β_2_m is more soluble than *h*β_2_m at pH 2.5**. (a) Final soluble protein concentration measured after incubation of *m*β_2_m or *h*β_2_m shown in *blue* and *black*, respectively. Samples were agitated for 14 days at pH 2.5 in 0 M (*closed* data points) or 0.3 M (*open* data points) NaCl at 37 °C. Error bars are the standard deviation of three replicate measurements and smaller than the data points if not visible. Data were fitted to determine the plateau regions, and hence the critical concentration (C_s_) (see Materials and Methods for details). (b) The C_s_ is plotted alongside the lag time of fibril growth under the same experimental conditions. Data for *m*β_2_m (C_s_ and lag time) are shown as > 1400 μM and > 60 hours, respectively, as no aggregation was detected under the conditions used. (c) The final soluble protein concentration for *m*β_2_m, *m*β_2_m(H7), *h*β_2_m and hβ_2_m(M7) incubated at pH 2.5 with 0.3 M NaCl are plotted. (d) The C_s_ values plotted alongside the lag time of fibril growth under the same experimental conditions.

To investigate how solubility contributes to the lag time of amyloid formation for *m*β_2_m, *m*β_2_m(H7), *h*β_2_m and *h*β_2_m(H7) the dependence of the lag times of fibril growth on ionic strength and the solubility of each protein was measured in the different conditions used. The critical concentration (C_s_) is the protein concentration at which phase separation occurs, thus any protein molecules above this concentration will, eventually, become insoluble [11], [25]. Many proteins can remain soluble above their critical concentration. Such supersaturated proteins are kinetically, but not thermodynamically, stable [31], [34]. The higher the protein concentration is above the critical concentration, the greater the driving force for aggregation [11], [25], [31]. Therefore the C_s_ of fibril formation can be used as a measure of solubility and reports on the driving force of aggregation. To determine the C_s_ for the different proteins studied in 0 M or 0.3 M NaCl, each protein was incubated for 14 days (with agitation) at pH 2.5 in the absence or presence of 0.3 M NaCl at 37 °C and soluble protein was then separated from insoluble protein by centrifugation (Materials and Methods). These experiments revealed that *m*β_2_m is substantially more soluble than its human counterpart in the absence of NaCl (Fig. 5a). Indeed, in the absence of NaCl *m*β_2_m remains ≥ 90 % soluble even at a protein concentration of 1.4 mM (Fig. 5a inset). The C_s_ of *m*β_2_m in 0 M NaCl is > 1.4 mM, compared with only 11 ± 4 μM in 0.3 M NaCl (Fig. 5b, Table 1). By contrast, *h*β_2_m is substantially less soluble than *m*β_2_m at both ionic strengths (Fig. 5b, Table 1). These results correlate with the marked difference in the lag times of the two proteins in the different solution condition used.

To explore the effect of the FG loop on protein solubility the critical concentrations of the chimeric proteins were also measured and compared with the corresponding values for wild-type *m*β_2_m and *h*β_2_m in 0.3 M NaCl (Fig. 5c and d). At this ionic strength there is a clear relationship between lag time and the C_s_ of each protein (Fig. 5d, Table 1). *m*β_2_m has a higher C_s_ and a longer lag time than *m*β_2_m(H7) (C_s_ values of 11.0 ± 4.3 μM and 3.4 ± 0.8 μM, respectively). *h*β_2_m and *h*β_2_m(M7) have C_s_ values 2.9 ± 0.4 μM and 4.3 ± 0.5 μM, respectively in 0.3 M NaCl, compared with 2.7 ± 0.5 μM for *h*β2m in 0 M NaCl. Nonetheless, these proteins have lag times that are 1.7- and 2.5-fold longer, respectively, than *h*β_2_m in 0 M NaCl (Fig. 5d, Table 1). This lack of correlation between C_s_ and lag time could be due to the *h*β_2_m sequence being at its optimal solubility for amyloid formation and, therefore, other effects start to dominate the lag time of amyloid formation, such as non-specific amorphous aggregation or specific ion binding [5], [6], [22], [38], [40].

## Discussion

### Solubility and amyloidogenicity

Previous work by Ivanova *et al.* attributed the low amyloidogenicity of *m*β_2_m to sequence differences in the FG-loop [4]. The results presented here concur with this view and reveal that this difference in amyloid propensity results predominantly from the effect of the FG-loop on protein solubility (Fig. 5). The acid unfolded states of *h*β_2_m and *m*β_2_m are both disordered (SI Fig. 1), therefore, it is unlikely that structural differences alone could explain the different amyloid propensities of the two sequences. The results thus highlight the critical role of protein solubility in determining the aggregation propensities of these two sequences.

**Fig. 6:**
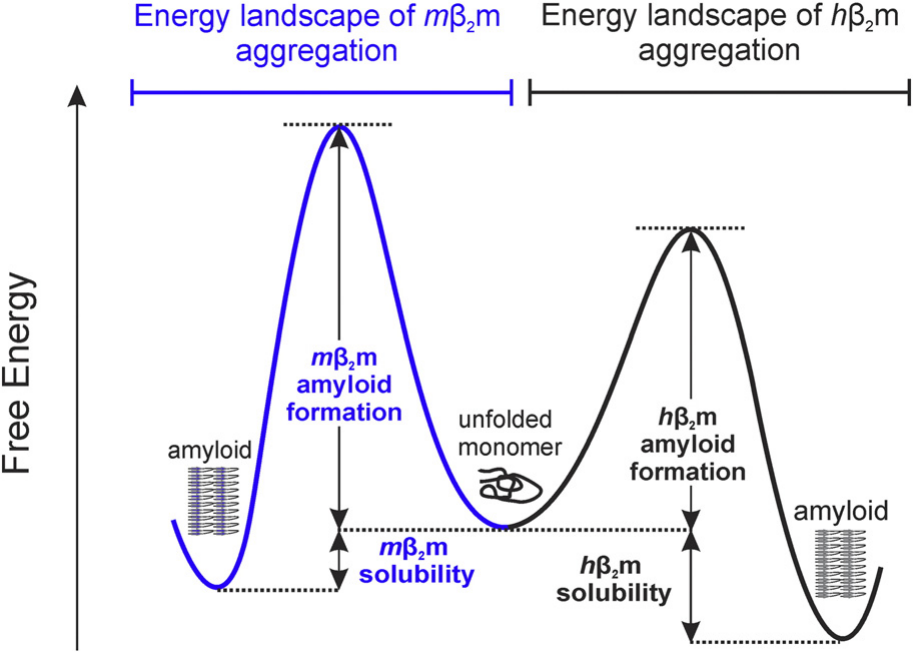
**Conceptual schematic energy landscape of β_2_m aggregation at low pH**. The solubility of a protein depends on the free energy difference between its monomeric and aggregated states. Formation of amyloid and amorphous aggregates (the aggregation propensity) depends on the activation free energy barrier between monomer and aggregate/amyloid and is therefore kinetically governed. The reaction could be more complicated than shown here since amorphous aggregates can be formed on-pathway or in competition with amyloid aggregates. In addition, each of the free energy differences will depend on the protein sequence (including point mutations and/or other larger sequence changes such as the *h*β_2_m/*m*β_2_m chimeras), pH, solution conditions (i.e. salt concentration) and protein concentration. The diagram should thus be used for conceptual purposes to show that *h*β_2_m has a greater amyloid propensity and lower solubility compared with *m*β_2_m.

Previous research has linked the aggregation of acid denatured *h*β_2_m to its solubility [20], and work by Goto & colleagues has proposed a phase diagram for ordered assembly of acid unfolded *h*β_2_m into amyloid fibrils, suggesting that protein solubility has an important role in aggregation and in the nucleation mechanism [26], [40]. Routledge *et al.* previously showed that they were able to better predict the aggregation rates of different *h*β_2_m variants at pH 2.5 by taking NMR relaxation properties (a measure of conformational dynamics and/or intermolecular interaction) into account [37]. Here we show that by increasing the ionic strength of the solution and unfolding the protein at low pH, *m*β_2_m is able to form amyloid-like fibrils. Yet *m*β_2_m does not aggregate in the absence of 0.3 M NaCl because the C_s_ of the protein is > 1.4 mM at lower ionic strengths. Subtle changes in the protein sequence and solution conditions, thus, can have dramatic effects on solubility and, in turn, on the lag time of amyloid formation. Interestingly the sequence in the native E-strand of *h*β_2_m has been shown to be critical for determining the rate of amyloid formation in the acid unfolded state, with the remainder of the sequence having little effect on the rate of amyloid formation [36], [37], [42]. These residues form only 11 out of a total 70 residues in the core of *h*β_2_m amyloid fibrils, which span residues G18-Q88 [43]. Therefore, while the sequence of the E-strand is solely important for determining the kinetics of aggregation, it is not necessarily central to thermodynamic stability of the fibril architecture. This example demonstrates that a greater understanding of the interplay between protein sequence and the driving force of aggregation that causes a metastable supersaturated protein to aggregate (Fig. 6).

### Consequences for amyloid formation in general

The FG loop region of *m*β_2_m is predicted to be more soluble than its *h*β_2_m equivalent by the CamSol intrinsic protein solubility prediction algorithm (Fig. 1c) [11], but to have only a modest effect on the aggregation/amyloid propensity of the sequences (Fig. 1d-h). In contrast, our results show this region has a significant effect on the lag time of amyloid formation. In seminal work by Chiti *et al.* the effects of amino acid substitutions on the rates of aggregation of the denatured sate of human acylphosphatase (AcP) were investigated [44]. The authors showed that the aggregation rates could be correlated with three physiochemical properties of the protein sequence; hydrophobicity, secondary structure propensity and charge [44]. These properties together are able to predict the trends in aggregation rates observed for disease-relevant peptides and natively unfolded proteins such as amylin, Aβ-peptide, tau and ∝-synuclein [44]. These observations were the foundation for Zyggregator, an algorithm which aims to predict the absolute aggregation rate for a given protein sequence [45]. Importantly these rates are predicted for proteins above their C_s_ and represent the elongation rate of amyloid fibrils rather than the nucleation rate or lag time [14], [45]. Other prediction algorithms aim to find the most amyloid-prone or aggregation-prone regions within a sequence, but are not able to predict rates of nucleation or elongation [12], [13], [15], [16].

Rousseau *et al.* have analysed the proteomes of 28 different organisms through the TANGO aggregation prediction algorithm [46]. This algorithm balances factors such as hydrophobicity, hydrogen bonds, secondary structure propensities and charge to predict sequence segments with high aggregation propensity [16]. Their results showed there is a selective pressure against aggregation-prone sequences, but also that when aggregation-prone regions are present there is a strong evolutionary pressure towards “gate-keeper” residues within the flanking regions [46]. Protein aggregation into amorphous or ordered aggregates is kinetically controlled, whilst solubility is thermodynamically governed (Fig. 6). However, there is an unavoidable correlation between aggregation rate and solubility given that similar physiochemical properties of the amino acid sequence govern each process [11]. These gate-keeper residues include proline, arginine and lysine which increase solubility and their incorporations in the flanking regions of aggregation-prone sequences reduces aggregation propensity and amyloid formation [46]. There are twenty-two residues that are different between *m*β_2_m and *h*β_2_m (Fig. 1b). Of these residues, ten are charge swapping amino acid substitutions. Seven of the ten are charged in *h*β_2_m and substituted with neutral residues in *m*β_2_m. One of the ten is a charge reversal, and the remaining two residues are neutral in *h*β_2_m but charged in *m*β_2_m. Interestingly, these two residues are in the FG-loop region, and only one would be charged at pH 2.5. There are also four additional proline residues in the *m*β_2_m sequence when compared with *h*β_2_m sequence, which may play a role in protecting the sequence from aggregation into amyloid. Although these prolines would be predicted to reduce amyloid propensity, they are spread throughout the sequence, which may explain why *m*β_2_m sequence is still able to adopt an amyloid structure when conditions are favourable.

The sequence differences in the FG-loop have a significant effect on the solubility of *m*β_2_m and *h*β_2_m, however this region alone is insufficient to fully confer the difference in solubility between *m*β_2_m and *h*β_2_m suggesting that other parts of the β_2_m sequence contribute to the overall solubility of the sequence. The 4-residue flanking regions either side of the E-strand are identical in both proteins and hence differences in the residues that flank the most aggregation-prone regions cannot be responsible for the difference in aggregation of *m*β_2_m and *h*β_2_m. Together, the results indicate that the entire protein sequence must be considered to understand the solubility and amyloidogenicity of a protein sequence, even for an unfolded protein.

The effects of protein solubility on amyloid formation *in vitro* have been highlighted recently in several systems. The flanking regions of polyglutamine repeat sequences can have a dramatic effect on solubility, affecting aggregation rates, as well as the nucleation mechanism [25]. The H50Q variant of α-synuclein has been shown to have decreased solubility which correlates with the decreased lag time of the protein compared with the wild-type sequence [24]. Experiments by Goto and colleagues have revealed that sonication can alter protein solubility at the air water interface and hence aid the initiation of amyloid formation [40]. These studies, together with the work presented here, highlight the importance of protein solubility in determining the rates of protein aggregation and reveal how differences in experimental design (i.e. surface area of the air water interface, vessel material, method of agitation, salt concentration, type of salt used) and subtle differences in sequence can affect the observed lag times of amyloid formation such that a protein such as *mβ*_2_m can escape from aggregation at mM concentration and low ionic strength.

Human and mouse β_2_m provide a particularly striking example in which solubility determines amyloid formation. The discrepancy between the dramatic difference in the rates of aggregation of *h*β_2_m and *m*β_2_m, with the relatively subtle differences in the predicted protein solubility (Fig. 1c) and aggregation propensity (Fig. 1d-h), highlights the need for a greater understanding of how sequence alters protein solubility and how amino acid substitutions modulate solubility in a sequence- and region-dependent manner. Here we have shown that a protein that is very soluble and yet contains sequences with high amyloid propensity (e.g. the E-strand of *m*β_2_m), does not aggregate in 0 M NaCl since it contains solubilising amino acid substitutions, including residues in the FG-loop, compared with *h*β_2_m. The solubility (i.e. the C_s_) is the driving force for aggregation and a key determinant of the lag time of amyloid formation of these unfolded proteins. Nonetheless, the lag time is a largely underutilised parameter for testing amyloid prediction algorithms. This was recently noted by Hall *et al.* who theorised that an understanding of protein solubility and how this effects the competition between amyloid and amorphous aggregation mechanisms may be informative for understanding the cause and age of onset of amyloid disease [22]. Since most proteins are on the edge of solubility [29], and many are already supersaturated in the cell [31], an enhanced ability to predict C_s_ accurately, and the effects of environmental factors in modulating solubility, could enable us to elucidate the tipping point at which a soluble protein will become aggregation-prone and could cause disease. Detailed investigation of protein solubility, and the effects of sequence and experimental conditions on C_s_ and the lag time of aggregation such as presented here for *h*β_2_m, *m*β_2_m and their chimeras, will be needed for many protein sequences in order to derive such an understanding.

## Materials and Methods

### Protein preparation

The synthetic *E*.*coli* codon optimised genes (Eurofins Genomics) for the chimeric proteins *h*β_2_m(M7) and *m*β_2_m(M7) were removed from the pEX-A vector with *Hin*dIII and *Nde*I and ligated into the pET 23a plasmid (Novagen) cut with the same enzymes. All variants of β_2_m were expressed and prepared as described previously [47], with the modification that anion-exchange purification buffers were used at pH 8.5 rather than pH 7.0 and gel filtration was performed in 10 mM sodium phosphate (pH 8.5) rather than 25 mM Tris HCl (pH 8.0) [47].

### Fibril growth experiments

For all experiments lyophilised protein was dissolved in water, sterile filtered (0.22 μM pore size, Millipore) and diluted to 50 μM in fibril growth buffer (25 mM sodium phosphate / 25 mM sodium acetate, pH 2.5). Additional NaCl was added as indicated. Fibril growth was monitored in Corning® 96-well polystyrene microtitre plates sealed with clear polyolefin sealing film (STARLAB) for seven days at 37 °C with agitation (200 rpm) using 100 μl per sample. Seeded reactions were performed using 10% seeds (w/w), in which fibrils were fragmented to create seeds by stirring at 1000 rpm as described previously [39]. Fibril growth was assessed by measuring the fluorescence of ThT (10 μM) (excitation 440 nm, emission 480 nm) using a Fluorostar Optima, BMG Labtech plate reader at 37 °C (50 readings/well). Fluorescence intensity is shown corrected for the signal of ThT in buffer alone and all data were normalised to the final ThT signal. The resulting curves were used to determine the lag times and apparent rates of elongation. The lag time was obtained by fitting a straight line to the steepest part of the slope of the growth phase (approximately 30% to 70% of the maximum amplitude), and the time at which this line intersected the baseline was taken as the lag time [37]. Synthetic peptides were supplied by Peptide Protein Research Ltd as a pure lyophilised powder and were dissolved in 100% DMSO. The stock solutions were then diluted into the fibril growth buffer. A final peptide concentration of 100 μM was used with a final concentration of ≤ 5% (v/v) DMSO.

### Circular dichroism

CD experiments were performed on a Chirascan plus spectrometer (Applied PhotoPhysics), with a bandwidth of 1 nm, scan speed of 20 nm min^− 1^, step size of 1 nm and a path length of 1 mm. An average of 4 scans was used for the final spectra. Spectra were recorded using a protein concentration of 0.2 mg/ml at 25 °C. Protein samples were incubated after acidification at 37 °C for at least 20 minutes and the CD spectra were then measured.

### ANS binding

The fluorescence emission spectra of ANS (Sigma-Aldrich) in the presence or absence of protein were recorded on a Photon Technology International (PTI) QM-1 spectrofluorimeter at 37 °C. Protein samples (10 μM) were prepared and diluted into the relevant buffer or solution that also contained ANS (250 μM final concentration). The fluorescence of ANS was determined immediately using an excitation wavelength of 389 nm and fluorescence emission was collected between 400 and 600 nm using slit widths of 5 nm. The fluorescence emission of ANS in buffer alone was then used to normalise spectra from different buffer conditions.

### Negative-stain EM

Carbon-coated copper grids were prepared by the application of a thin layer of formvar with an overlay of carbon. Protein samples (10 μl) were applied drop wise. The grid was thin dried with filter paper before washing with 2 x 10 μl of deionised water, blotting with filter paper between steps. Negative staining was achieved by the addition of 10 μl of 2% (w/v) uranyl acetate, which was subsequently blotted with filter paper. A second addition of 10 μl of 2% (w/v) uranyl acetate was allowed to stain for 30 seconds before blotting on filter paper. Micrographs were recorded on a *JOEL* JEM-1400 electron microscope equipped with a Gatan Orius camera.

### Sedimentation velocity AUC

Sedimentation velocity experiments were carried out at 25 °C using a Beckman Optima XL-I analytical ultracentrifuge (Beckman, Palo Alto, CA) using an An-60 Ti rotor with conventional aluminium double-sector centrepieces with a rotor speed of 48,000 rpm. Samples of 50 μM protein were prepared by exchanging the sample into the relevant buffer by overnight dialysis at 4 °C. Radial absorbance scans at 280 nm were collected at 300 s intervals and the data were analysed using SEDFIT [48].

### NMR spectroscopy

All NMR experiments were carried out at 25 °C using Varian Unity Inova spectrometers operating at ^1^H frequency of 500 MHz. Protein samples were prepared in buffer or water with 10 % (v/v) D_2_O. Gradient enhanced ^1^H-^15^N HSQC spectra were acquired using 160 complex points and 16 scans per increment with spectral widths of 8511 Hz and 1800 Hz in the ^1^H and ^15^N dimensions, respectively. Watergate solvent suppression was used, and all NMR data were processed using NMRPipe and analysed in NMRView [49], [50].

### Solubility assay

Proteins were incubated at 50 μM in fibril growth buffer (25 mM sodium phosphate/25 mM sodium acetate, pH 2.5) with the addition of NaCl as indicated. Samples were incubated at 200 rpm for two weeks at 37 °C (500 μl in a 1.5 ml Eppendorf tube), before being centrifuged at 14,000 *g* for 30 minutes on a bench top centrifuge. The soluble protein was measured by Bicinchoninic Acid (BCA) assay, or absorbance at 280 nm. An extinction coefficient of 18575 M^− 1^ cm^− 1^ was used to measure the protein concentration of *m*β_2_m in the absence of NaCl. A microBCA (Pierce Biotechnology Inc) assay was performed to measure the supernatant concentrations of the other samples due to their low critical concentrations. The “test tube” protocol for the microBCA assay was followed, but reagent volumes were reduced to measure 100 μl protein samples. To determine the C_s_, curves were fitted to Eq. (1), where X is the initial protein concentration, Cs is the plateau concentration and K is the protein concentration value at half height of the curve.

(1) $$\mathrm{Signal}=\frac{\left(C_{s}\right)X}{K+X}$$

## Figures and Tables

**Table 1:** – Lag times of amyloid formation and solubility of different β_2_m variants

	0 M NaCl		0.3 M NaCl	
Protein	Lag-time^a^ (Hours)	C_s_ (μM)^b^	Lag-time^a^ (Hours)	C_s_ (μM)^b^
*h*β_2_m	5.5 ± 1.1	2.7 ± 0.5	9.2 ± 0.6	2.9 ± 0.4
*m*β_2_m	> 60	> 1400	30.1 ± 4.5	11.0 ± 4.3
*h*β_2_m(M7)	4.2 ± 1.1	n.d.	13.7 ± 1.5	4.3 ± 0.5
*m*β_2_m(H7)	> 60	n.d.	4.1 ± 0.5	3.4 ± 0.8

**^a^**Lag time determined by fitting a straight line to the steepest part of the slope of the growth phase (see Material and Methods).

**^b^**Critical concentration determined by agitated incubation of various concentrations of protein and measuring the protein concentration of the supernatant after 2 weeks (see Materials and Methods). n.d., not determined.
