# Supplementary material for: Comparison of the aggregation of homologous β2-microglobulin variants reveals protein solubility as a key determinant of amyloid formation
Source: J Mol Biol. 2016 Feb 13;428(3):631–43. doi: 10.1016/j.jmb.2016.01.009 (PMC4773402; doi:10.1016/j.jmb.2016.01.009)
Supplement: Supplementary file 1 — Supplementary Figures [file mmc1.pdf]

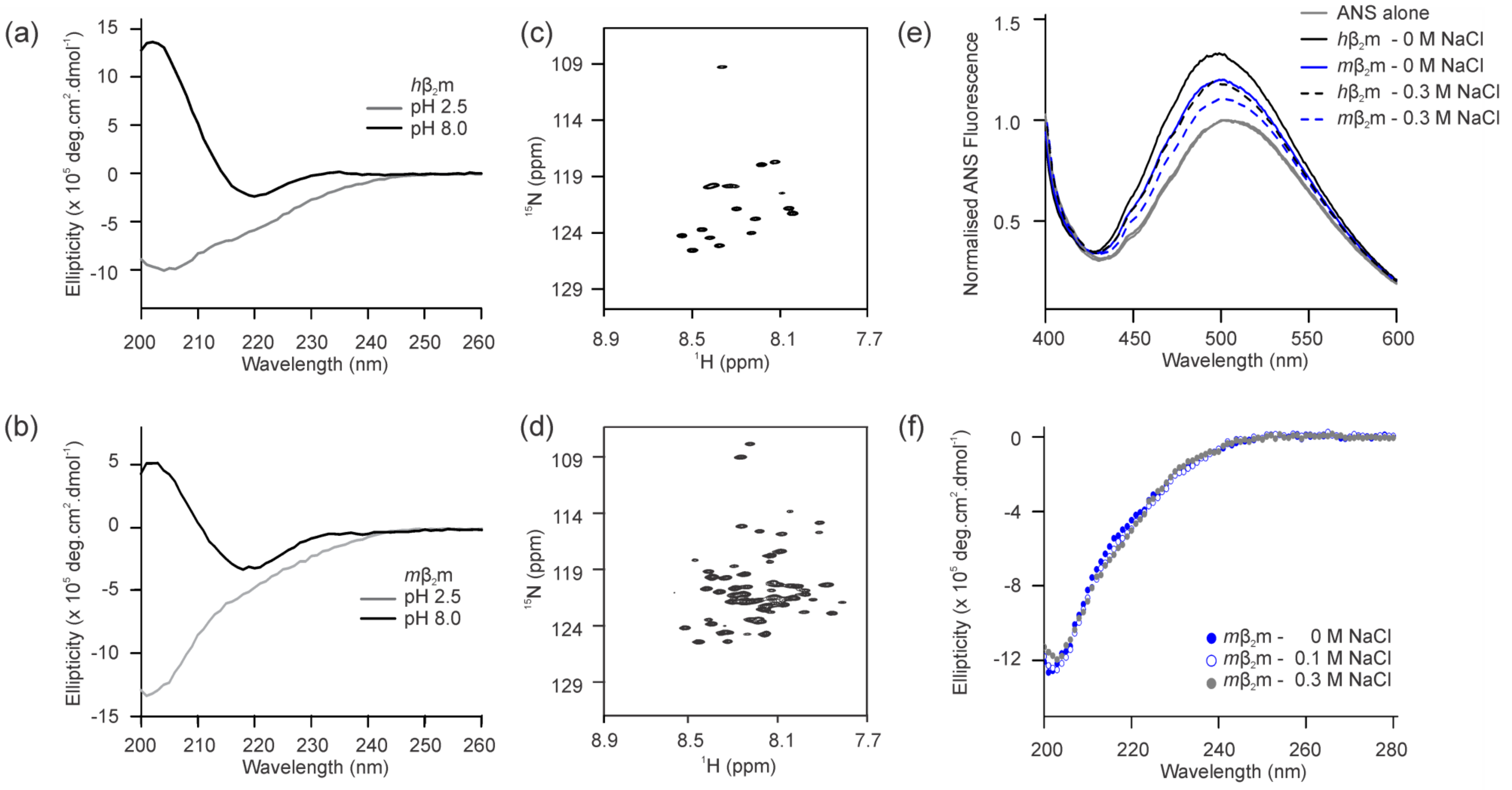

SI Figure 1 – Structural characterisation of acid unfolded β_2m

Far UV CD spectra of folded and acid-unfolded (a) *h*β_2m and (*b*) mβ_2m taken in 10 mM phosphate (pH 8.0) or fibril growth buffer (pH 2.5) at 25 °C (Materials and Methods). The ^{1}H-^{15}N HSQC spectrum of 150 µM (c) *h*β_2m and (d) *m*β_2m acquired in 25 mM sodium phosphate/25mM sodium acetate (pH 2.5) at 25 °C. (e) ANS fluorescence of *h*β_2m (black) and *m*β_2m (blue) taken at pH 2.5 in 0 M NaCl (solid line) or in the presence of 0.3 M NaCl (dotted line). The spectra were normalised to the spectrum of ANS in buffer alone. (f) Far UV CD spectra of *m*β_2m in pH 2.5 fibril growth buffer with 0 M (solid blue points), 0.1 M (hollow blue data points) or 0.3 M NaCl (solid grey data points).

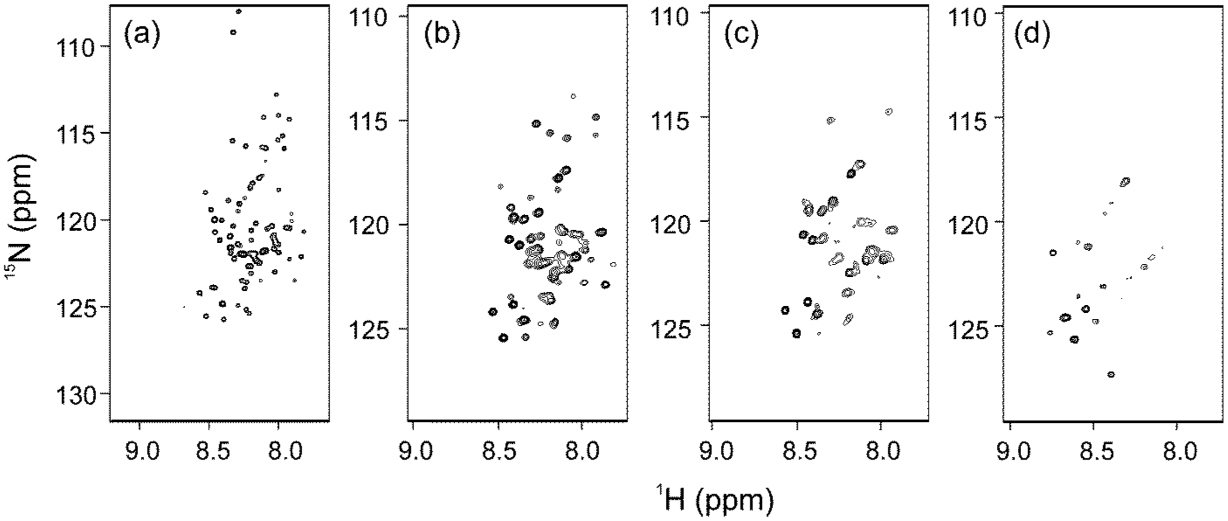

SI Figure 2 - increasing ionic strength induces NMR line broadening

The ^{1}H-^{15}N HSQC spectrum of 150 µM acid unfolded *m*β$_2$m performed at 25 °C in (a) pH 2.5 H_2O, (b) 25 mM sodium phosphate/25 mM sodium acetate (pH 2.5), (c) 25 mM sodium phosphate/25 mM sodium acetate plus 0.3 M NaCl and (d) 25 mM sodium phosphate/25 mM sodium acetate plus 0.8 M NaCl. Spectra are contoured at the same level for comparison.

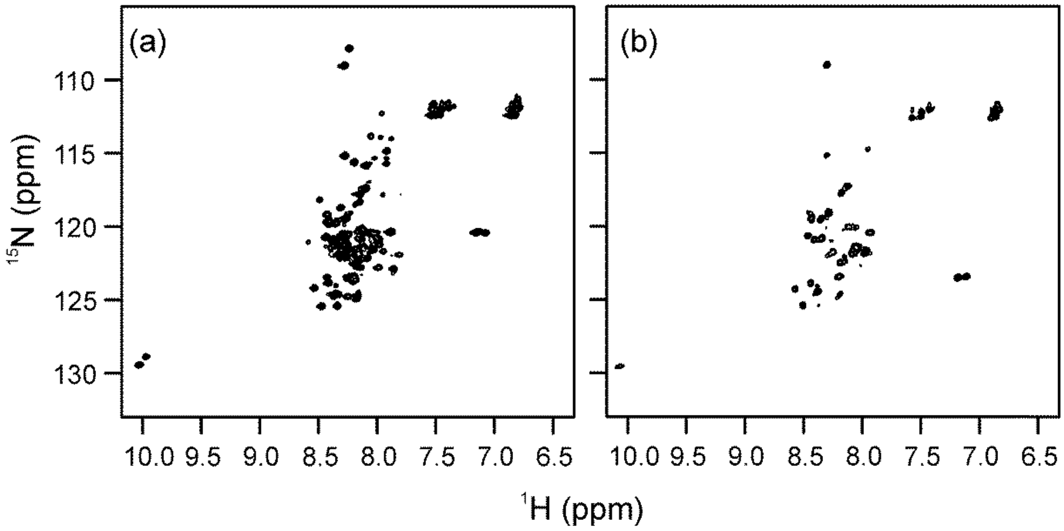

SI Figure 3 – Tuning artefacts are not the origin of line broadening in high salt
The ^{1}H-^{15}N HSQC spectrum of 150 µM acid unfolded $m\beta_2m$ performed at 25 °C in fibril growth buffer with 0.8 M NaCl and (a) 8 M urea or (b) 0 M urea. Spectra are contoured at the same level for comparison.

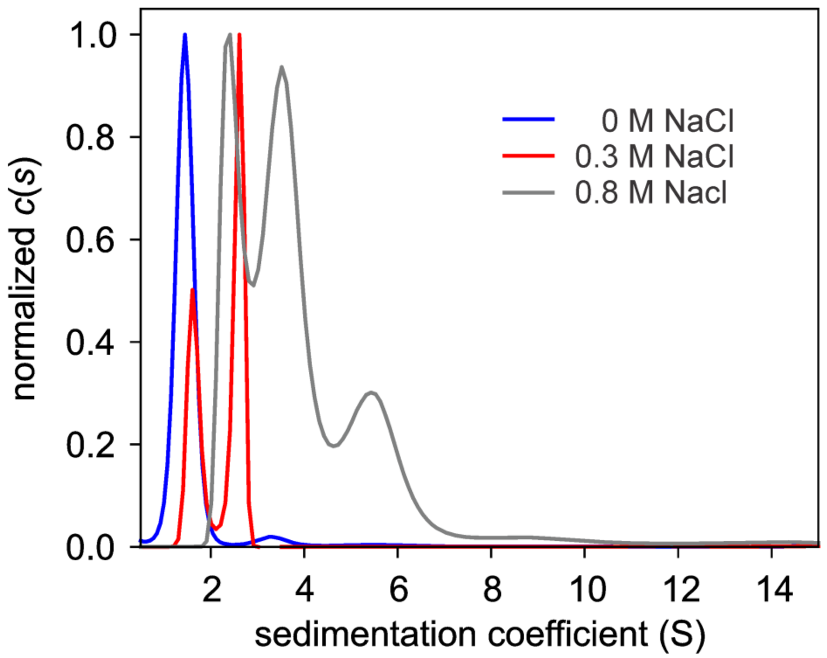

SI Figure 4 – Salt induced protein-protein interactions

Sedimentation velocity analytical ultracentrifugation of 50 μM *m*β$_2$m performed at 48,000 rpm at 25°C in 25 mM sodium phosphate/25mM sodium acetate buffer (pH 2.5) with 0 M NaCl, 0.3 M NaCl or 0.8 M NaCl, shown in *blue*, *red* and *grey*, respectively.

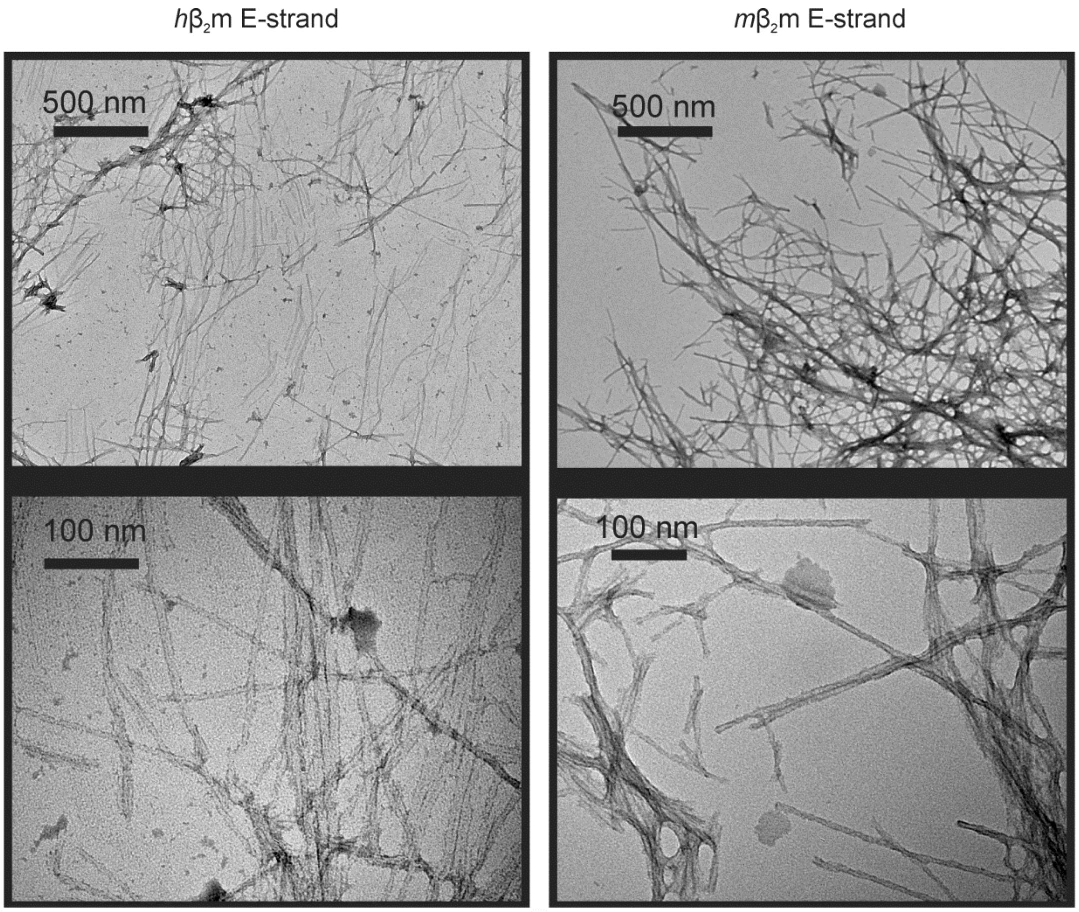

SI Figure 5

Negative stain EM images of fibrils grown from synthetic peptides corresponding to the E-strand of *h*β_2m (left panel) and *m*β_2m (right panel). 100 μM of the *h*β_2m (Ac-DWSFYLLYYTEF-NH_2) and *m*β_2m (Ac-DWSFYILAHTEF-NH_2) peptide were incubated for 1 week in pH 2.5 fibril growth buffer agitated at 200 rpm at 37°C.
